# β-Elemene Piperazine Derivatives Induce Apoptosis in Human Leukemia Cells through Downregulation of c-FLIP and Generation of ROS

**DOI:** 10.1371/journal.pone.0015843

**Published:** 2011-01-25

**Authors:** Zhiying Yu, Rui Wang, Liying Xu, Siwei Xie, Jinhua Dong, Yongkui Jing

**Affiliations:** 1 School of Life Science and Biopharmaceutics, Shenyang Pharmaceutical University, Shenyang, China; 2 Key Laboratory of Structure-Based Drug Design and Discovery, Ministry of Education, Shenyang Pharmaceutical University, Shenyang, China; 3 Department of Medicine, The Tisch Cancer Institute, Mount Sinai School of Medicine, New York, New York, United States of America; University of Hong Kong, Hong Kong

## Abstract

β-Elemene is an active component of the herb medicine *Curcuma Wenyujin* with reported antitumor activity. To improve its antitumor ability, five novel piperazine derivatives of β-elemene, 13-(3-methyl-1-piperazinyl)-β-elemene (DX1), 13-(cis-3,5-dimethyl-1-piperazinyl)-β-elemene (DX2), 13-(4-ethyl-1-piperazinyl)-β-elemene (DX3), 13-(4-isopropyl-1-piperazinyl)-β-elemene (DX4) and 13-piperazinyl-β-elemene (DX5), were synthesized. The antiproliferative and apoptotic effects of these derivatives were determined in human leukemia HL-60, NB4, K562 and HP100-1 cells. DX1, DX2 and DX5, which contain a secondary amino moiety, were more active in inhibiting cell growth and in inducing apoptosis than DX3 and DX4. The apoptosis induction ability of DX1 was associated with the generation of hydrogen peroxide (H_2_O_2_), a decrease of mitochondrial membrane potential (MMP), and the activation of caspase-8. Pretreatment with the antioxidants *N*-acetylcysteine and catalase completely blocked DX1-induced H_2_O_2_ production, but only partially its activation of caspase-8 and induction of apoptosis. HL-60 cells were more sensitive than its H_2_O_2_-resistant subclone HP100-1 cells to DX1-induced apoptosis. The activation of caspase-8 by these compounds was correlated with the decrease in the levels of cellular FLICE-inhibitory protein (c-FLIP). The proteasome inhibitor MG-132 augmented the decrease in c-FLIP levels and apoptosis induced by these derivatives. FADD- and caspase-8-deficient Jurkat subclones have a decreased response to DX1-induced apoptosis. Our data indicate that these novel β-elemene piperazine derivatives induce apoptosis through the decrease in c-FLIP levels and the production of H_2_O_2_ which leads to activation of both death receptor- and mitochondrial-mediated apoptotic pathways.

## Introduction

β-Elemene is one of the active components in the essential oil of *Curcuma Wenyujin*, a traditional Chinese herb medicine. β-Elemene has been shown to inhibit tumor cell growth *in vitro* and *in vivo* and has been put into clinical trials in cancer patients in China [1], [2], [3]. However, due to its poor water-solubility and the requirement of high concentrations to reach therapeutic effects, the efficacy of β-elemene in cancer treatment is limited. To improve its activity we have synthesized a series of β-elemene derivatives which contain piperazine, morpholine, tetrahydropyrrole, thiophenylethylamine, or cyclohexamine groups [4]. Among these derivatives, 13,14-bis(cis-3,5-dimethyl-1-piperazinyl)-β-elemene was found to be one of the most potent agents in inhibiting the growth of leukemia cells [4].

Based on these previous observations, five novel β-elemene derivatives with substitution of one different piperazinyl group, 13-(3-methyl-1-piperazinyl)-β-elemene (DX1), 13-(cis-3,5-dimethyl-1-piperazinyl )-β-elemene (DX2), 13-(4-ethyl-1-piperazinyl)-β-elemene (DX3), 13-(4-isopropyl-1-piperazinyl)-β-elemene (DX4) and 13-piperazinyl-β-elemene (DX5), were synthesized. The abilities of these compounds to inhibit cell growth and to induce apoptosis as well as their mechanisms of apoptosis induction were investigated in several human leukemia cell lines. All of the five compounds inhibited cell growth with IG_50_s less than 10 µM. Compounds with a secondary amino moiety (DX1, DX2 and DX5) were more potent than compounds without a secondary amino moiety (DX3 and DX4) in inducing apoptosis. Mechanism studies of apoptosis induction revealed that both the mitochondrial- and the death receptor-mediated apoptotic pathways were involved. The mitochondrial apoptotic pathway is activated due to cleavage of Bid by activated caspase-8 and by the production of reactive oxygen species (ROS). The role of ROS in the apoptosis induction by these compounds was investigated using antioxidants and a H_2_O_2_-resistant cell line. The activation of caspase-8 was investigated by assessing levels of the death receptors and the cellular FLICE-inhibitory protein (c-FLIP). Jurkat cells deficient of Fas-associated death domain protein (FADD) and caspase-8 were used to evaluate the role of caspase-8 activation in the apoptosis induction due to these compounds. Our data suggest that these novel β-elemene derivatives induce apoptosis through both the death receptor- and the mitochondrial-mediated apoptotic pathways due to down-regulation of c-FLIP protein and the production of ROS, respectively.

## Materials and Methods

### Reagents

DX1-DX5 were synthesized using similar methods to those that we reported previously [4] and were prepared as maleates. The chemical structures were characterized with IR spectroscopy, ^1^H-NMR spectroscopy, mass spectrometry, and elemental analyses. *N*-acetylcysteine (NAC), ethidium bromide (EB), acridine orange (AO), propidium iodide (PI), catalase (CAT) and TRAIL receptor 2/Fc chimera (TRAIL R2/Fc) were purchased from Sigma Chemical Co. (St. Louis, MO). Rhodamine-123 (Rh123) and 5,6-carboxy-2′,7′-dichlorodihydrofluorescein diacetate (DCFH-DA) were obtained from Molecular Probes (Eugene, OR). Antibody to poly-(ADP-ribose)-polymerase was obtained from Boehringer Mannheim (Indianapolis, IN), antibodies to caspase-3, caspase-8 and CD95L were from BD Biosciences (San Diego, CA), antibodies to DR4, DR5 and c-FLIP were from Alexis Biochemicals (San Diego, CA), antibodies to Bid, NOK-1, CD95 and β-actin were from Santa Cruz Biotechology, Inc. (Santa Cruz, CA), and antibodies to caspase-9 and TRAIL were from Cell Signaling Technology (Beverly, MA).

#### Cell lines

HL-60, NB4 and K562 cells were cultured in RPMI-1640 medium supplemented with 100 units/mL penicillin, 100 µg/mL streptomycin, 1 mmol/L *L*-glutamine, and 10% (v/v) heat-inactivated fetal bovine serum (FBS) as we described previously [5]. HP100-1 (obtained from the Japanese Cell Bank) is a H_2_O_2_-resistant subclone of HL-60 cells [6]. Jurkat subclones, A3, FADD-deficient I 9.1, and caspase-8-deficient I 9.2 were obtained from ATCC and were cultured in RPMI 1640 supplemented with 10% heat-inactivated FBS [7].

#### Cell growth inhibition

Cells were seeded at 1×10^5^ cells/mL and incubated with various concentrations of β-elemene piperazine derivatives for 72 h. Total cell number was determined with the aid of a hemocytometer and cell viability was estimated by trypan blue exclusion [8].

#### MTT assay

Two hundred µL of HL-60 cells at a density of 1×10^5^/ml containing various concentrations of DX1 were plated in each well of 96-well plates. The cells were cultured for 12 and 24 h at 37°C. 3-(4, 5-Dimethylthiazol-2-yl)-2, 5-diphenyltetrazolium bromide (MTT) solution (50 µl of 2 mg/ml) was added per well and the cultures were continued for an additional 4 h. The medium was aspirated after centrifugation at 1000 RPM for 10 min, the cells were dissolved in 100 µl DMSO, and the optical density (OD) at 570 nm was determined in each well with a 96-well plate reader. The cytotoxicity was calculated as ODt/ODc×100%. ODc represents the OD of the control group and ODt represents the OD of the treated group.

#### Quantitation of apoptotic cells

Apoptotic cells were determined by morphologic observation, fluorescence-activated cell sorting (FACS) analysis after staining with PI, and Annexin V/PI. [8]. For morphologic observation, cells were stained with AO and EB and assessed by fluorescence microscopy as described previously [8]. Briefly, 1 µL of stock solution containing 100 µg/mL AO and 100 µg/mL EB was added to 25 µL of cell suspension. EB-negative cells with nuclear shrinkage, blebbing and apoptotic bodies were counted as apoptotic cells. The percentage of apoptotic cells was calculated after observing a total of 300 cells. For FACS analysis with PI staining, cells were fixed with ice-cold 70% ethanol at a density of 1×10^5^ cells/mL and treated with 200 µg/mL RNase for 30 min at 37°C. PI was then added to the solution at a final concentration of 50 µg/mL and the DNA content was quantitated by flow cytometry (Becton Dickinson, San Jose, CA) with an excitation wavelength of 488 nm and an emission wavelength of 625 nm. Data were analyzed using CELLQuest (Becton Dickinson) software. For the annexin V staining analysis, 10^5^ cells were washed twice with PBS, then labeled with annexin V-FITC and PI in binding buffer according to the instructions provided by the manufacturer in the Annexin V-FITC Apoptosis Detection Kit (Oncogene, Cambridge, MA). The fluorescent signals of FITC and PI were detected at 518 nm and at 625 nm, respectively, with a flow cytometer. Data were analyzed using CELLQuest (Becton Dickinson) software.

#### Quantification of DNA fragmentation

DNA fragmentation was quantified as described previously [5]. Cells were harvested by centrifugation, and the pellets were suspended in lysis buffer containing 15 mmol/L Tris·HCl, 20 mmol/L EDTA, 0.5% Triton X-100, pH 8.0. After 30 min on ice, samples were centrifuged at 14,000*g* for 30 min, and cellular DNA was extracted. Electrophoresis was performed in 1% agarose gel in 40 mmol/L Tris-acetate buffer (pH 7.4) at 50 V. After electrophoresis, DNA was visualized by EB staining.

#### Determination of intracellular H_2_O_2_

Intracellular H_2_O_2_ was monitored by flow cytometry after staining with DCFH-DA. In the present study, cells were labeled with 5 µM DCFH-DA for 1 h and then treated with or without β-elemene piperazine derivatives at 37°C for various times. After washing with phosphate buffer saline (PBS), cells were analyzed by flow cytometry with excitation and emission wavelengths of 495 and 525 nm, respectively. Cells treated with 100 µM H_2_O_2_ for 1 h were used as a positive control [5].

#### Measurement of MMP

MMP was assessed by the retention of Rh123, a membrane-permeable fluorescent cationic dye that is selectively taken up by mitochondria. Its fluorescence intensity is proportional to MMP levels. Cells treated with β-elemene piperazine derivatives for various times were collected and incubated with 0.3 µg/mL Rh123 in the dark for 20 min at room temperature. After washing with PBS, the cells were analyzed by flow cytometry with excitation and emission wavelengths of 495 and 535 nm, respectively.

#### Western blot analysis

Protein extracts (50 µg) prepared with RIPA lysis buffer [50 mmol/L Tris-HCl, 150 mmol/L NaCl, 0.1% sodium dodecyl sulfate (SDS), 1% NP-40, 0.5% sodium deoxycholate, 1 mmol/L phenylmethyl sulfonyl fluoride (PMSF), 100 µmol/L leupeptin, and 2 µg/mL aprotinin, PH 8.0] were separated on an 8% or 12% SDS-polyacrylamide gel and transferred to nitrocellulose membranes. The membranes were stained with 0.2% Ponceau S red to assure equal protein loading and transfer. After blocking with 5% nonfat milk, the membranes were incubated with a specific antibody overnight at 4°C. Immunocomplexes were visualized by ECL Western Blotting Detection reagents (Amersahm Biosciences, UK). Protein contents in the lysate were determined by the Bradford protein binding assay [9].

#### RNA interference

FLIP_S/L_ siRNA (sc-35388) and a negative control siRNA (sc-37007) were purchased from Santa Cruz Biotechnology, Inc. siRNA was transfected into the K562 cells by electroporation (Amaxa, Gaithersburg, MD) following the manufacturer's instructions. Briefly, 2×10^6^ cells were electroporated in 100 µL nucleofector solution (Amaxa Reagent V) containing 30 pmol of each siRNA using the preselected Amaxa Program T-016. siRNA transfected cells were plated in a 6-well plate with 2 mL supplemented RPMI-1640 medium with 10% FBS for 15 h and subsequently further treated with or without 10 µM DX1 for 24 h. Cells treated with or without DX1 were harvested for Western blotting analysis.

### Statistics

The Student's t-test (Microsoft Excel, Microsoft Corporation, Seattle, WA) was performed to determine the significance between groups. A *P*-value of less than 0.05 (*P*<0.05) was considered to be statistically significant.

## Results

### β-elemene piperazine derivatives inhibit cell growth and induce cytotoxicity in HL-60 cells

**Figure 1:**
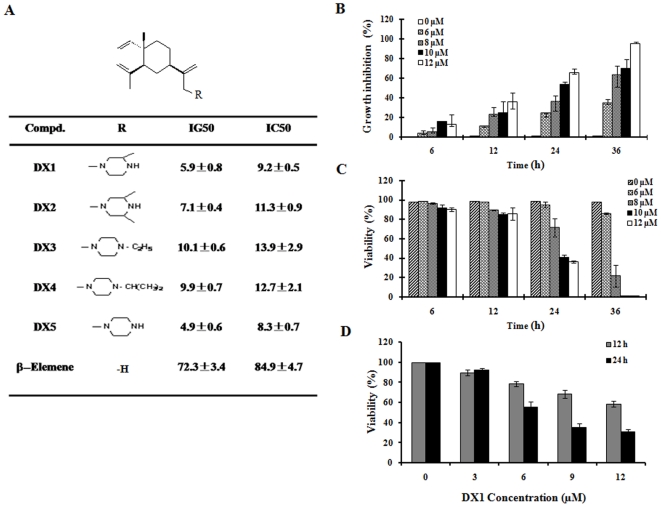
The structures, the antiproliferative and cytotoxic effects of β-elemene piperazine derivatives in HL-60 cells. (**A**) **The substitutions of β-elemene piperazine derivatives and their IG_50_s and IC_50_s.** HL-60 cells were treated with various concentrations of these compounds for 4 days. The cell number and viability were determined. The drug concentrations that inhibited half of the cell growth (IG_50_) or that killed half of the cells (IC_50_) were calculated. The data shown are the mean plus SE of three independent experiments. (**B**) **Antiproliferative effects of DX1.** (**C**) **Cytotoxicity of DX1 measured by trypan blue exclusion.** (**D**) **Cytotoxicity of DX1 measured by the MTT assay.** HL-60 cells were treated with the indicated concentrations of DX1 for the indicated times. Cell growth inhibition (**B**) and cytotoxicity (**C&D**) were determined as described in Materials and Methods. The data shown are the mean plus SE of three independent experiments.

HL-60 cells were treated with various concentrations of β-elemene piperazine derivatives for 4 days. The levels of cell numbers and viable cells were determined. The concentrations which inhibited 50% of cell growth (IG_50_) and killed half of the cells (IC_50_) were calculated. DX1, DX2 and DX5 were more potent than DX3 and DX4 in inhibiting cell growth and in inducing cytotoxicity (Fig. 1A). All five derivatives were more effective than β-elemene in inhibiting HL-60 cell growth (Fig. 1A). The time- and dose-dependent effects of DX1 on cell growth and viability of HL-60 cells are shown in Fig. 1B, 1C, 1D. DX1 at a concentration of 10 µM killed about 60% of the cells after 24 h of treatment as determined by trypan blue exclusion (Fig. 1C) and MTT assay (Fig. 1D). DX1 at a concentration of 10 µM killed all of the cells after 36 h of treatment determined by trypan blue exclusion (Fig. 1C).

### DX1 decreases MMP, increases H_2_O_2_ production and induces apoptosis in HL-60 cells

**Figure 2:**
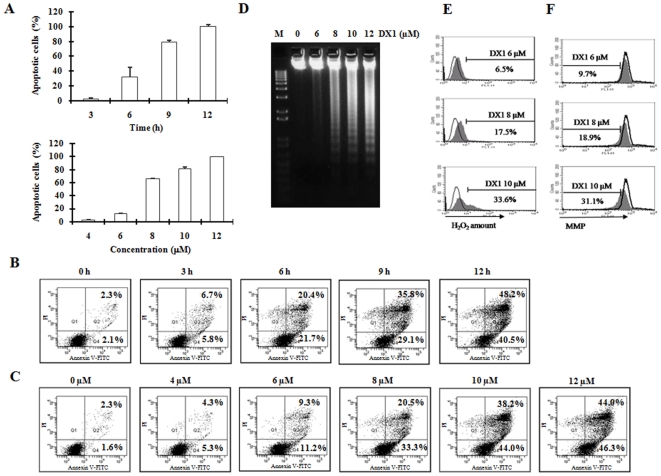
Apoptosis induction, ROS production and the decrease in MMP by DX1 treatment in HL-60 cells. (**A**) **Apoptotic cell levels determined by morphological observation after AO/EB staining.** HL-60 cells were treated with DX1 at the indicated concentrations for 10 h or treated with DX1 at 10 µM for the indicated times. The percentage of apoptotic cells was determined morphologically using a fluorescence microscope after staining with AO and EB. The data shown are the mean plus SE of three independent experiments. (**B**) **Time-dependent and (C) Dose-dependent apoptosis induction due DX1 treatment determined by FACS analysis after staining with Annexin V/PI.** HL-60 cells were treated with DX1 at 10 µM for the indicated times (**B**) or treated with DX1 at the indicated concentrations for 10 h (**C**). The percentage of apoptotic cells was determined by FACS after staining with Annexin V. (**D**) **Fragmented DNA.** HL-60 cells were treated with DX1 at the indicated concentrations for 10 h and total DNA was isolated. The levels of fragmented DNA was determined using staining with EB after electrophoresis in agarose gel. M, DNA marker. (**E**) **H_2_O_2_ production.** HL-60 cells were labeled with 5 µM DCFH-DA fluorescent probe for 1 h and then treated with or without DX1 at the indicated concentrations for 8 h. Oxidized DCF levels were analyzed using FACS as described in Materials and Methods. Open peaks, untreated cells; shaded peaks, DX1-treated cells with the labeled concentrations. (**F**) **MMP.** HL-60 cells were treated with DX1 at the indicated concentrations for 8 h. Alterations of MMP were determined according to changes in fluorescence density upon Rhodamine 123 loading as described in Materials and Methods. Open peaks, untreated cells; shaded peaks, treated cells. The peak shift to left indicates a loss of MMP.

To determine whether the cytotoxicity of DX1 is due to induction of apoptosis, apoptotic cells were determined based on morphologic examination after staining with AO and EB in HL-60 cells. DX1 induced apoptosis in a time- and dose-dependent pattern (Fig. 2A). Apoptotic cells were detected in cultures after treatment with 10 µM DX1 for 6, 9 and 12 h and after treatment with 6, 8, 10 and 12 µM DX1 for 10 h. DX1 (12 µM ) induced 97% of cells to undergo apoptosis after 10 h of treatment. The apoptosis induction ability of DX1 was further confirmed by FACS analysis after staining with Annexin V/PI (Fig. 2B, 2C) and determination of fragmented DNA (Fig. 2D).

H_2_O_2_ production was measured in HL-60 cells after DX1 treatment using a H_2_O_2_-sensitive fluorescent probe, DCFH-DA. The effect of DX1 on MMP was determined by flow cytometry using the cationic dye Rh123. DX1 induced a dose-dependent production of H_2_O_2_ and a decrease in MMP (Fig. 2E & 2F). Time course studies revealed that DX1 increased the amount of H_2_O_2_ as early as 2 h after beginning treatment (data not shown). These data suggest that the mitochondrial-apoptotic pathway, probably due to H_2_O_2_ production, is involved in the apoptosis induction by DX1 treatment.

### Antioxidants NAC and CAT partially inhibit DX1-induced apoptosis

**Figure 3:**
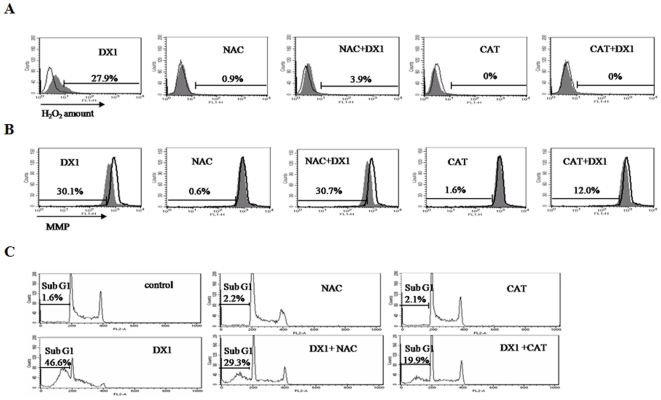
The antioxidants NAC and CAT block H_2_O_2_ production, but not on the loss of MMP by DX1 treatment in HL-60 cells. (**A**) **H_2_O_2_ levels; (B) MMP; (C) Apoptotic cells.** HL-60 cells were pretreated with or without NAC (10 mM) or CAT (500 U/ml) for 4 h followed by treatment with 10 µM DX1 for 8 h. The levels of H_2_O_2_ and MMP were measured using FACS as described in Materials and Methods. Open peaks, untreated cells; shaded peaks, DX1-treated cells with the labeled agents (A and B). The percentage of apoptotic cells was determined by flow cytometric analysis after PI staining as described in Materials and Methods.

To evaluate the role of ROS accumulation in DX1-induced apoptosis, we investigated the effects of antioxidants NAC and CAT on DX1-induced apoptosis and on the decrease in MMP. Pretreatment with antioxidants NAC and CAT prevented DX1-induced H_2_O_2_ accumulation (Fig. 3A). However, pretreatment with NAC and CAT only partially blocked the loss of MMP and apoptosis due to DX1 treatment (Fig. 3B and 3C). These data suggest that H_2_O_2_ production only plays a partial role in the process of DX1-induced apoptosis.

### DX1 activates caspase-3, -8 and -9 and decreases the levels of c-FLIP protein

**Figure 4:**
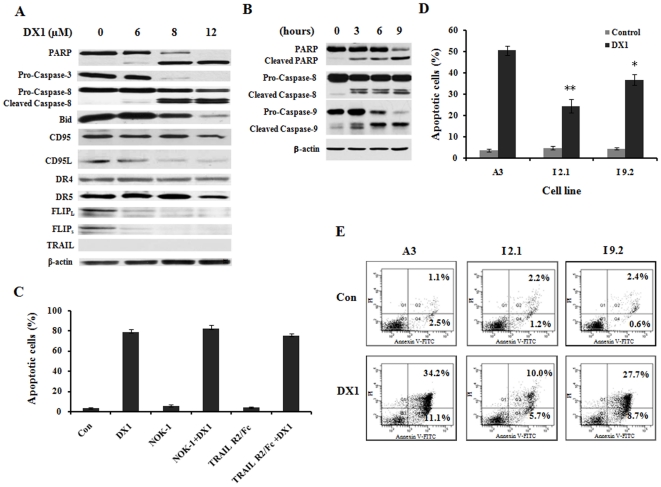
Caspase-8 activation and its role in DX1-treated cells. (**A**) **Western blot analyses of protein levels.** HL-60 cells were treated with DX1 at the indicated concentrations for 10 h. The levels of each protein as indicated were detected using a specific antibody as described in Materials and Methods. (**B**) **Time-dependent cleavage of caspase-8 and -9.** HL-60 cells were treated with DX1 at 10 µM for the indicated times. (**C**) **The effects of NOK-1 and TRAIL-R2/Fc on DX1-induced apoptosis.** HL-60 cells were pretreated with 1 µg/ml NOK-1 or 1 µg/ml TRAIL-R2/Fc for 4 h followed by treatment of 10 µM DX1 for 10 h. The percentage of apoptotic cells was determined morphologically using a fluorescence microscope after staining with AO and EB. The data shown are the mean plus SE of three independent experiments. (**D & E**) **Apoptosis induction of DX1 in FADD- and caspase-8- deficient Jurkat subclones.** FADD-deficient clone I 2.1, caspase-8-deficient clone I 9.2 and parental clone A3 were treated with DX1 at the indicated concentrations for 10 h. The percentage of apoptotic cells was determined morphologically using a fluorescence microscope after staining with AO/EB (**D**) and by FACS after staining with Annexin V/PI (**E**). The data shown (**D**) are the mean plus SE of three independent experiments. * *P*<0.05, I 9.2 cells treated with DX1 compared to A3 cells treated with DX1; ** *P*<0.01, I 2.1 cells treated with DX1 compared to A3 cells treated with DX1.

To explore the apoptotic machinery, the protein levels of Bid, caspase-3, caspase-8, caspase-9, CD95, CD95L, c-FLIP, DR4, DR5, PARP and TRAIL were determined in HL-60 cells after treatment with several concentrations of DX1 using Western blotting analyses. DX1 treatment induced a dose-dependent reduction in the levels of pro-caspase-3, pro-caspase-8, Bid and c-FLIP (Fig. 4A). DX1 treatment induced cleavage of caspase-8 and PARP (Fig. 4A). Activation of caspase-3 leads to the cleavage of PARP. Caspase-8 can activate caspase-3 directly or indirectly through cleavage of Bid followed by the activation of caspase-9. Time-dependent induction of caspase-8 and caspase-9 cleavage was determined (Fig. 4B). The cleaved fragments of both caspase-8 and caspase-9 were detected after 3 h of treatment with DX1 (Fig. 4B). These data suggest that both death receptor- and mitochondrial-mediated apoptotic pathways are involved in DX1-induced apoptosis. Caspase-8 activation by aggregated or ligated death receptors is mediated through FADD [10]. The protein levels of death receptors, DR4, DR5, CD95 and their ligands TRAIL and CD95L were not increased in those cells treated with DX1 (Fig. 4A). Neither CD95L neutralizing antibody NOK-1 nor human recombinant DR5 (TRAIL R2)/Fc chimera protein blocked the apoptosis induction by DX1 treatment (Fig. 4C). The activation of caspase-8 is inhibited by c-FLIP [11]. Both the long form c-FLIP_L_ and the short form c-FLIP_S_ were reduced after treatment with DX1 (Fig. 4A). Jurkat subclones, I 2.1 cells, lacking FADD, and I 9.2 cells, lacking caspase-8, were less sensitive to DX1-induced apoptosis compared to their parental subclone, A3 as measured by observation of morphological changes after AO/EB staining (Fig. 4D) and by FACS analysis after Annexin V/PI staining (Fig. 4E). These data suggest that the activation of caspase-8 plays a partial role in DX1-induced apoptosis. The activation of caspase-8 by DX1 treatment is probably mediated by decreases in c-FLIP levels, but not by the induction of death receptor levels or by the ligation of the death receptors.

### MG-132 enhances DX1-induced apoptosis in HL-60 cells

**Figure 5:**
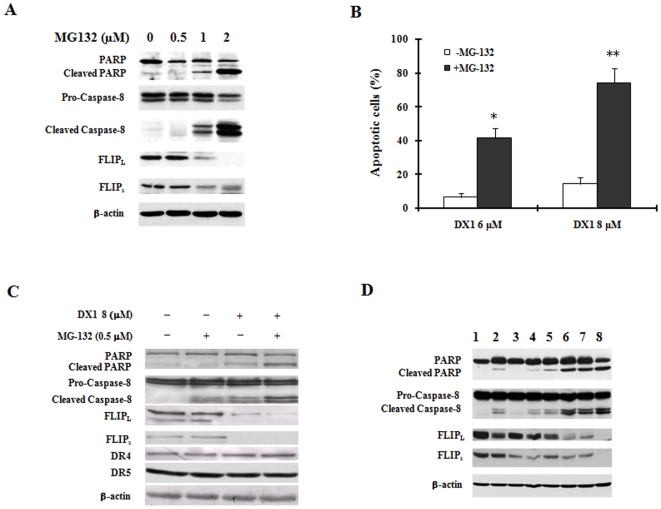
MG-132 enhanced apoptosis induction and caspase-8 cleavage due to DX1 treatment in HL-60 cells. (**A**) **Western blot analysis of PARP, caspase-8 and c-FLIP levels in HL-60 cells treated with MG-132.** HL-60 cells were treated with DMSO (control), MG-132 at the indicated concentrations for 10 h. (B) **Apoptotic cells determined morphologically.** HL-60 cells were treated with DMSO (control), MG-132 0.5 µM, DX1 8 µM or MG132 plus DX1 for 10 h. The percentage of apoptotic cells was determined by morphological observation after AO/EB staining as described in Materials and Methods. * *P*<0.05 and ** *P*<0.01 compared to cells treated without MG132. (**C**) **Western blot analysis of PARP, caspase-8, c-FLIP, DR4 and DR5 proteins.** HL-60 cells were treated with DMSO (control), MG-132 0.5 µM, DX1 8 µM or MG132 plus DX1 for 10 h. (**D**) **Time-dependent decrease of c-FLIP levels due to treatment by MG-132 plus DX1 in HL-60 cells.** Lane 1, control cells; Lane 2, cells treated with 0.5 µM MG-132 for 9 h; Lane 3, cells treated with 8 µM DX1 for 3 h; Lane 4, cells treated with 8 µM DX1 plus 0.5 µM MG-132 for 3 h; Lane 4, cells treated with 8 µM DX1 for 6 h; Lane 6, cells treated with 8 µM DX1 plus 0.5 µM MG-132 for 6 h; Lane 7, cells treated with 8 µM DX1 for 9 h; Lane 8, cells treated with 8 µM DX1 plus 0.5 µM MG-132 for 9 h. The levels of each protein were detected using specific antibodies as described in Materials and Methods.

It has been shown that c-FLIP is degraded by a proteasome-mediated pathway and that the proteasome inhibitor MG-132 blocked c-FLIP degradation in cells treated with several agents [12], [13]. Moreover, it also has been reported that MG-132 decreased the levels of c-FLIP and sensitized TRAIL-induced apoptosis [14], [15]. The effects of MG-132 on the apoptosis induction and on c-FLIP levels were determined in HL-60 cells by Western blotting analysis. HL-60 cells were responsive to MG-132-induced apoptosis determined by PARP cleavage. The PARP cleavage was observed after treatment with MG-132 at a concentrations higher than 1 µM. MG132 decreased the levels of both long and short forms of c-FLIP (Fig. 5A). MG-132 at 0.5 µM alone did not induce apoptosis or c-FLIP decrease and this concentration was selected to test the effects of MG-132 on DX1-induced apoptosis and c-FLIP downregulation (Fig. 5B, 5C). MG132 at 0.5 µM by itself apparently did not induce apoptosis. DX1 at 8 µM induced apoptosis in 14.6% of cells (Fig. 5B), whereas combination treatment of MG-132 with DX1 induced apoptosis in 74.6% of the cells (Fig. 5B). Correlated with this apoptosis induction, the cleavage of PARP and caspase-8 as well as decreases in c-FLIP levels were enhanced by the combination treatment (Fig. 5C). Time-dependent results revealed that MG-132 enhanced DX1-induced decrease in c-FLIP levels after 6 h treatment (Fig. 5D).

### DX1-induced apoptosis is correlated with the downregulation of c-FLIP in NB4, HP100-1 and K562 cells

**Figure 6:**
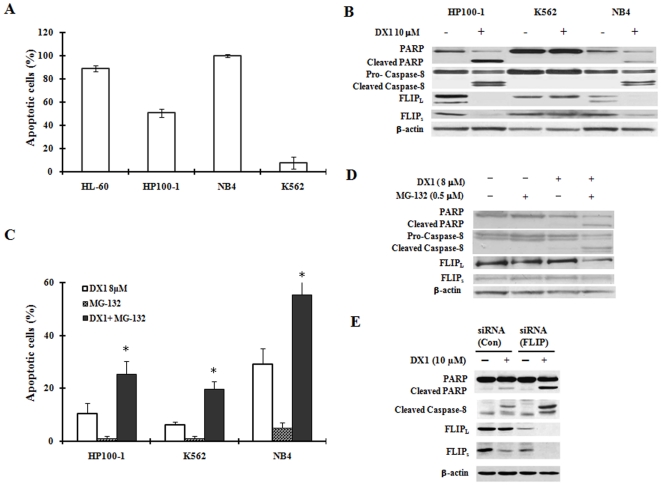
Apoptosis induction, caspase-8 cleavage, and the decrease in c-FLIP levels due to DX1 treatment in NB4, HP100-1 and K562 cells. (**A**) **Apoptotic cells.** HL-60, HP100-1, NB4 and K562 cells were treated with 10 µM DX1 for 9 h. The percentage of apoptotic cells was determined by morphological observation after AO/EB staining as described in Materials and Methods. (**B**) **The protein levels and cleavage of PARP, caspase-8 and c-FLIP.** NB4, HP100-1 and K562 cells were treated with or without 10 µM DX1 for 10 h. The levels of each protein were detected using specific antibodies as described in Materials and Methods. (**C**) **MG-132 enhancement of DX1-induced apoptosis.** Cells were treated with DMSO (control), MG-132 0.5 µM, DX1 8 µM or MG132 plus DX1 for 10 h (NB4 and HP100-1 cells) or for 36 h (K562 cells). The percentage of apoptotic cells was determined morphologically after AO/EB staining as described in Materials and Methods. * *P*<0.05 compared to cells treated with DX1 alone. (**D**) **MG-132 enhancement of DX1-induced cleavage of PARP and caspase-8 in K562 cells.** K562 cells were treated with DMSO (control), MG-132 0.5 µM, DX1 8 µM or MG-132 plus DX1 for 36 h. The levels of each protein were detected using specific antibodies as described in Materials and Methods. (**E**) **The influence of c-FLIP siRNA on DX1-induced cleavage of PARP and caspase-8 in K562 cells.** K562 cells were incubated with control siRNA or c-FLIP siRNA for 15 h and then treated with or without 10 µM DX1 for 24 h. The levels of each protein were deteced using specific antibodies as described in Materials and Methods.

The abilities of DX-1 to induce apoptosis and to downregulate c-FLIP were further investigated in three additional leukemia cell lines. NB4 cells were as sensitive as HL-60 cells to DX1-induced apoptosis (Fig. 6A). HP100-1 cells were less sensitive to DX1-induced apoptosis compared to HL-60 and NB4 cells (Fig. 6A). K562 cells did not respond to DX1-induced apoptosis at 10 µM treatment for 10 h (Fig. 6A). However, DX1 induced apoptosis in K562 cells at increased concentrations and prolonged incubation times (data not shown). The cleavage of PARP and caspase-8 as well as the decrease in the c-FLIP levels occurred in NB4 and HP100-1 cells, but not in K562 cells (Fig. 6B). Therefore, these processes correlate with the sensitivity to DX1-induced apoptosis at 10 µM treatment in these three cell lines (Fig. 6A). MG-132 enhanced DX1-induced apoptosis at 8 µM in NB4 and HP100-1 cells at 12 h, but in K562 cells only at treatment times of 36 h or longer (Fig. 6C). MG-132 plus DX1 augmented to induce cleavage of both PARP and caspase-8 and to decrease the levels of c-FLIP in K562 cells after 36 h of treatment (Fig. 6D). To further determine the role of c-FLIP in DX1-induced apoptosis, the c-FLIP levels were knocked-down using siRNA. Augmented PARP cleavage was observed in K562 cells transfected with c-FLIP siRNA (Fig. 6E).

### DX1, DX2 and DX5 are more active than DX3 and DX4 in inducing apoptosis and in decreasing c-FLIP levels in HL-60 cells

**Figure 7:**
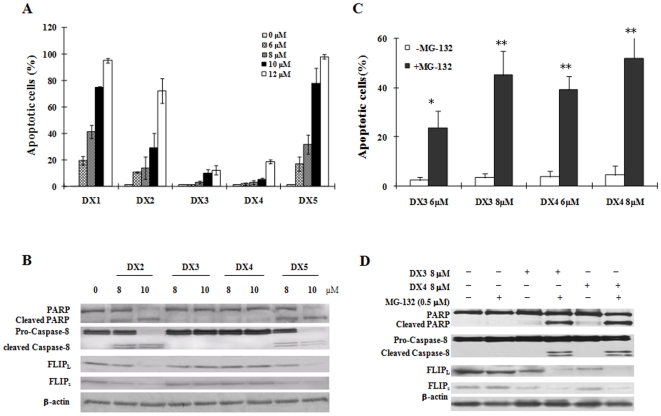
Apoptosis induction, caspase-8 cleavage, and the reduction of c-FLIP levels by treatment with DX2, DX3, DX4 and DX5 in HL-60 cells. (**A**) **Apoptotic cells.** HL-60 cells were treated with DX1, DX2, DX3, DX4 or DX5 at the indicated concentrations for 10 h. The percentage of apoptotic cells was determined using a fluorescence microscope after staining with AO and EB as described in Materials and Methods. The data shown are the mean plus SE of three independent experiments. (**B**) **The protein levels and cleavage of PARP, caspase-8 and c-FLIP.** HL-60 cells were treated with 8 or 10 µM of each compound for 10 h. The levels of each protein were detected using a specific antibody as described in Materials and Methods. (**C**) **MG-132 enhancement of DX3- and DX4-induced apoptosis.** HL-60 cells were treated with DMSO (control), MG-132 0.5 µM, DX3/DX4 or MG132 plus DX3/DX4 at the indicated concentrations for 10 h. The percentage of apoptotic cells was determined using a fluorescence microscope after staining with AO and EB as described in Materials and Methods. * *P*<0.05 and ** *P*<0.01 compared to cells treated without MG132. (**D**) **MG-132 enhancement of DX3- and DX4-induced cleavage of PARP and caspase-8 as well as the reduction in c-FLIP levels.** HL-60 cells were treated with DMSO (control), MG-132 0.5 µM, DX3 8 µM, DX4 8 µM, MG-132 plus DX3 or MG-132 plus DX4 for 10 h. The levels of each protein were detected using a specific antibody as described in Materials and Methods.

The apoptosis induction abilities of DX2, DX3, DX4 and DX5 were compared to that of DX1 in HL-60 cells. DX2 and DX5 had similar activities as that of DX1 in apoptosis induction. DX1, DX2 and DX5 at 12 µM induced more than 80% of HL-60 cells to undergo apoptosis after 10 h of treatment (Fig. 7A). DX3 and DX4 at 12 µM only induced less than 15% of cells to undergo apoptosis after 10 h of treatment (Fig. 7A). DX3 and DX4 at 12 µM induced higher percentages of apoptotic cells at prolonged incubation times (data not shown). At 10 µM, DX2 and DX5, but not DX3 or DX4, induced cleavage of PARP and caspase-8, and decreased the levels of c-FLIP (Fig. 7B). MG-132 plus DX3 or DX4 had augmented effects in inducing apoptosis and in decreasing c-FLIP levels compared to any of these agents alone (Fig. 7C&7D). DX1, DX2 and DX5 contain a secondary amino moiety (Fig. 1). The hydrogen at position 4 of piperazine was substituted by an ethyl and an isopropyl in DX3 and DX4, respectively (Fig. 1). Based on their abilities to induce apoptosis and to decrease c-FLIP levels, it seems that the secondary amino moiety is an important enhancing group for the biological activity of these β-elemene derivatives.

## Discussion

β-Elemene inhibits cell growth only at high concentrations. Previously we found that β-elemene substituted with a tryptophan methyl ester improved its antiproliferative effects and induced apoptosis in leukemia cells through a ROS-mediated pathway [16]. In this study, we found that β-elemene substituted with a piperazine was even more effective than β-elemene substituted with a tryptophan methyl ester in inducing apoptosis. These compounds acted through both ROS production and c-FLIP reduction. The IG50 of these β-elemene derivatives substituted with a piperazine was decreased to less than 10 µM (Fig. 1A). Based on the cell growth inhibition abilities of DX1 to DX5, the substitution with one (DX1) or two methyl groups (DX2) of piperazine does not evidently influence the antiproliferative and cytotoxic effects of these piperazine derivatives. However, replacement of the hydrogen of the C-4 of piperazine with an ethyl (DX3) or an isopropyl (DX4) decreases the antiproliferative and cytotoxic effects compared to those of DX1, DX2 and DX5 which have a hydrogen at C-4. DX1, DX2 and DX5 induced evident apoptosis at concentrations of 6–10 µM after treatment for 10 h (Fig. 7A). DX3 and DX4 induced apoptosis at higher concentrations and more prolonged incubation times (data not shown). These data suggest that the apoptosis induction of these compounds is an important mechanism for their ability to induce cytotoxicity.

DX1 was used to explore the mechanism of apoptosis induction. It is known that death receptor and mitochondrial apoptotic pathways play important roles in apoptosis induction due to chemotherapeutic agents [17], [18], [19]. Several agents have been found to induce the mitochondrial-mediated apoptotic pathway through the generation of ROS which decreases the MMP which then leads to activation of caspase-3 [5], [20]. DX1 increased the production of ROS, decreased MMP levels, and activated caspase-3 in HL-60 cells (Fig. 2). To investigate the role of ROS in DX1-induced apoptosis, antioxidants NAC and CAT were used. Although both NAC and CAT could prevent the ROS production due to DX1 treatment in HL-60 cells, they only had minimal effects in preventing cells from undergoing DX1-induced apoptosis (Fig. 3). Since both NAC and CAT only partially block DX1-induced decrease in MMP, it seems that the decrease in MMP after DX1 treatment, at least in part, is mediated through an ROS-independent pathway (Fig. 3). HP100-1 cells, which are resistant to H_2_O_2_ [6], [21], are responsive to DX1 treatment (Fig. 6A). These data suggest that ROS production only plays a partial role in DX1-induced apoptosis. Thus, the action of DX1 is different from that of N-(b-elemene-13-yl)tryptophan methyl ester which induced apoptosis only by increasing ROS [16].

The death receptor-mediated pathway can also lead to decreases in MMP through cleavage of BID due to activated caspase-8 [22]. It has been found that several agents could activate caspase-8 through increasing the levels of death receptors [23], [24], [25], [26]. DX1 treatment activated caspase-8 based on the determination of its cleavage and decrease in the levels of Bid (Fig. 4A). Therefore, the decrease in MMP levels by DX1 treatment could be partly due to cleaved Bid. Previously we have found that boswellic acids induced apoptosis by activated caspase-8 due to induction of DR4 and DR5 proteins [27], [28]. However, the levels of DR4, DR5 and CD95 as well as their ligands CD95L and TRAIL were not increased after DX1 treatment (Fig. 4A). Neither CD95L neutralizing antibody NOK-1 nor human recombinant DR5 (TRAIL R2)/Fc chimera protein was able to block DX1-induced apoptosis (Fig. 4C). These data suggest that DX1 activates caspase-8 through a pathway independent of the regulation of death receptor levels and their activation. c-FLIP has been found to inhibit the activation of caspase-8. Several other agents have been found to activate caspase-8 by decreasing the levels of c-FLIP [29], [30], [31]. The protein levels of both c-FLIP_L_ and c-FLIP_S_ were reduced by DX1 treatment in a concentration-dependent pattern that was correlated with the cleavage of both caspase-8 and PARP (Fig. 4A). DX1 treatment induced cleavage of caspase-8 and PARP and decreased c-FLIP levels in NB4 and HP100-1 cells, but not in K562 cells (Fig. 6B). DX2 and DX5 treatments decreased the levels of c-FLIP and induced the cleavage of caspase-8 and PARP in HL-60 cells (Fig. 7B). DX3 and DX4 treatment did not decrease the levels of c-FLIP nor induce the cleavage of caspase-8 and PARP in HL-60 cells (Fig. 7B). FADD-deficient and caspase-8-deficient Jurkat subclones were less sensitive to DX1-induced apoptosis (Fig. 4D, 4E). Silencing c-FLIP augmented DX1-induced apoptosis in K562 cells (Fig. 6E). These data suggest the activation of caspase-8 is probably mediated through downregulation of c-FLIP and that activated caspase-8 plays a partial, but pivotal, role in the apoptosis induction by these compounds.

c-FLIP is known to be regulated by a ubiquitin-proteasome mechanism, and several cancer therapeutic agents have been found to induce downregulation of c-FLIP through this mechanism [32], [33], [34]. MG-132 has been reported to inhibit the decrease of c-FLIP levels induced by several agents in cancer cells [13], [35], [36]. However, it also has been reported that MG-132 decreased c-FLIP levels and enhanced TRAIL-induced apoptosis in prostate cancer cells and chronic lymphocytic leukemia cells [14], [15]. The difference of MG-132 action on c-FLIP levels may be cell type- and/or agent-dependent. In HL-60 cells MG-132 alone induced apoptosis and decreased the levels of c-FLIP at a concentration higher than 1 µM (Fig. 5A). We examined the effects of DX1 on the levels of c-FLIP and apoptosis induction in the presence and absence of MG-132 at a lower concentration. MG-132, at a concentration of 0.5 µM, weakly decreased c-FLIP levels and augmented DX1 induction of apoptosis and its ability to decrease c-FLIP levels (Fig. 5C, 5D). Our data indicate that MG-132 enhances the effects of these DX compounds to induce apoptosis by decreasing c-FLIP levels in these leukemia cells. Since the concentrations of MG-132 used here are very low, it is possible that reduction of c-FLIP by MG-132 is independent of its ability of inhibiting proteasome activity. The mechanism of inducing downregulation of c-FLIP levels by DX and MG-132 in leukemia cell lines needs to be further studied.

In summary, the present study reports the apoptotic effects and the mechanisms of action of five novel β-elemene piperazine derivatives. They induce apoptosis through production of ROS and decrease in c-FLIP levels and, thus, activate both death receptor-mediated and mitochondrial-mediated apoptotic pathways.
